# Thoracic Endovascular Aortic Repair and Endovascular Aneurysm Repair Approaches for Managing Aortic Pathologies: A Retrospective Cohort Study

**DOI:** 10.3390/jcm13185450

**Published:** 2024-09-13

**Authors:** Badr Aljabri, Kaisor Iqbal, Tariq Alanezi, Mussaad Al-Salman, Talal Altuwaijri, Mohammed Yousef Aldossary, Ghadah A. Alarify, Leen S. Alhadlaq, Sarah A. Alhamlan, Sultan AlSheikh, Abdulmajeed Altoijry

**Affiliations:** 1 Division of Vascular Surgery, Department of Surgery, College of Medicine, King Saud University, Riyadh 11322, Saudi Arabia; baljabri@ksu.edu.sa (B.A.); kiqbal@ksu.edu.sa (K.I.); mussaad@ksu.edu.sa (M.A.-S.); taltuwaijri@ksu.edu.sa (T.A.); dr.mohd.aldossary@gmail.com (M.Y.A.); sualsheikh@ksu.edu.sa (S.A.); 2 College of Medicine, King Saud University, Riyadh 11322, Saudi Arabia; alanezitariq@gmail.com (T.A.); leenalhadlaq@gmail.com (L.S.A.); sarah.a.a.alhamlan@gmail.com (S.A.A.); 3 Division of Vascular Surgery, Department of Surgery, Dammam Medical Complex, Dammam 32245, Saudi Arabia

**Keywords:** aortic aneurysms, aneurysms, aortic dissection, endovascular procedures

## Abstract

**Background/Objectives**: Since thoracic endovascular aortic repair (TEVAR) and endovascular aneurysm repair (EVAR) are increasingly utilized, examining their outcomes and safety in real-world scenarios is crucial. This study investigated the management and outcomes of TEVAR and EVAR as alternatives to traditional open surgical repair for managing aortic pathologies. **Methods**: This was a retrospective cohort study. We analyzed the data from 59 consecutive patients who underwent TEVAR or EVAR between 2015 and 2022 at a single tertiary care center. The primary outcome was survival, and secondary outcomes were complications assessment, including re-intervention and occurrence of endoleaks. **Results**: TEVAR accounted for 47.5% of cases (n = 28), while EVAR comprised 52.5% (n = 31). Patients were mostly 61–70 years old (23.7%) and male (91.5%). Surgery indications differed, with aneurysmal repair being the prevalent indication for EVAR (90.3%, n = 28) and trauma being the main indication for TEVAR (67.9%, n = 19). Regarding the primary outcome, 11 patients (18.6%) died for various reasons; of those, 2 patients (3.4%) were determined to have died from vascular-related issues. Most patients (81.4%, n = 48) did not experience intraoperative complications. The most common intraoperative complications were endoleaks and access failure, each affecting 5.1% (n = 3) of patients. Re-intervention was necessary in 16.9% (n = 10) of cases, with endoleaks being the major indication (60%). Emergency intervention was more frequent in the TEVAR group *(p* = 0.013), resulting in significantly longer hospitalization (*p* = 0.012). **Conclusions**: Despite limitations, our analysis indicates a good safety profile with high success rates and a low incidence of adverse health outcomes and mortality in TEVAR/EVAR procedures. Nevertheless, the results emphasize the ongoing concern of endograft leaks, necessitating re-interventions.

## 1. Introduction

The aorta, comprising thoracic and abdominal components, is susceptible to several pathologies, including aortic aneurysms [1,2]. Over time, untreated aortic aneurysms can lead to progressive weakening of the aortic wall, which increases the risk of a catastrophic rupture. The mortality rate associated with ruptured aortic aneurysms is alarmingly high, ranging from 50% to 80% [3].

The management of these aneurysms is complex and varies depending on the size, symptoms, and patient-specific factors. Abdominal aortic aneurysms (AAAs) require surgical interventions when certain conditions are met. However, careful surveillance is necessary for diminutive asymptomatic aneurysms < 5.5 cm in size due to the hazards of medical intervention surpassing those of rupture [4]. Thoracic aortic aneurysms and AAAs have similar pathologies involving different aortic regions. The pathophysiology of thoracic aortic aneurysms and AAAs shares many similarities, despite their occurrence in different regions of the aorta. Both types of aneurysms are influenced by a significant overlap in risk factors, including male gender, advanced age, a history of smoking, as well as a family history of cardiovascular conditions such as coronary artery disease (CAD) and hypertension [1].

In recent years, the landscape of treatment for aortic aneurysms has undergone a significant transformation with the advent of endovascular techniques. Consequently, endovascular aneurysm repair (EVAR) for AAAs and thoracic endovascular aortic repair (TEVAR) for thoracic aortic aneurysms have emerged as popular alternatives to traditional open surgical repair (OSR). These minimally invasive procedures offer several advantages, including reduced perioperative morbidity, shorter hospital stays, and quicker recovery times. Furthermore, over the past decade, there have been significant advancements in stent-graft design, delivery systems, and image-based planning tools. These refinements have been accompanied by an expansion of indications, now encompassing patients with more challenging landing zones, such as larger aneurysms and those closer to the aortic arch [5]. As a result, EVAR and TEVAR have become increasingly preferred options for managing aortic aneurysms, particularly in patients who may be at higher risk for complications from OSR. Compared with OSR, for instance, TEVAR offers several benefits, including decreased morbidity, avoidance of thoracotomy or sternotomy incisions, elimination of aortic cross-clamping, reduced blood loss, and decreased risk of end-organ ischemia [6]. Furthermore, several randomized controlled trials and systematic reviews have emphasized endovascular benefits over OSR, including substantially reduced perioperative and 30-day mortality rates [7,8]. A comparative study assessing AAA treatment outcomes indicated that EVAR may yield superior short-term outcomes, whereas OSR may offer better long-term outcomes for middle- or high-risk patients [9].

Despite the growing adoption of endovascular techniques, evaluating their effectiveness and safety in real-world clinical settings remains essential. The outcomes of TEVAR and EVAR procedures can vary based on factors such as patient selection, procedural expertise, follow-up care, and other variables [5]. Given the critical nature of aortic aneurysms and the rapid advancements in vascular intervention techniques, this study seeks to assess the management strategies and outcomes associated with TEVAR and EVAR within the context of a tertiary care center.

## 2. Materials and Methods

### 2.1. Study Design, Setting, and Population

This retrospective cohort study was conducted at the Department of Vascular Surgery, King Khalid University Hospital, Riyadh, Saudi Arabia. Patients who underwent TEVAR or EVAR between January 2015 and December 2022 for various etiologies, such as aortic aneurysms, dissections, aortic ulcers, and aortic fistulas, were included. Patients who underwent OSR were excluded. The primary outcome assessed was the mortality rate. The secondary outcomes were complication assessment, including re-intervention and the occurrence of endoleaks.

### 2.2. Data Source and Variables

The STrengthening the Reporting of OBservational studies in Epidemiology (STROBE) checklist for cross-sectional studies was implemented [10]. Various patient parameters such as age, sex, body mass index (BMI), and smoking status were extracted from electronic medical records (EMRs). Additionally, the surgery indications, operation priority (elective or emergency), surgery type (TEVAR, EVAR, Chimney), stents used (Medtronic, Gore, Bentley), and femoral access approach (cutdown, percutaneous) were recorded.

Additionally, we recorded intraoperative complications (through EMRs), type of anesthesia administered, aneurysmal diameter (cm), procedure duration (min), estimated blood loss (mL), blood urea nitrogen (BUN), and creatinine levels before and after intervention. Furthermore, crucial patient outcome and follow-up data, including transfer to the intensive care unit (ICU), mortality, cause of death, vascular-related postoperative complications, rate and type of postoperative endoleak, re-intervention rate, indications for re-intervention, number of days in the ICU, and the total hospital stay, were analyzed. Patients were followed up in the outpatient setting after discharge. Readmission and loss to follow-up were recorded and analyzed.

#### 2.2.1. Power Analysis

To compare the outcomes of TEVAR (n = 28) and EVAR (n = 31), the post hoc test of power (1 − β err prob) yielded 0.917, which exceeded the minimum of 0.80. Hence, considering the achieved power for this statistical test with two-tailed parameters and a medium effect size with alpha = 0.05, the sample size of TEVAR (n = 28) and EVAR (n = 31) cases were considered sufficient.

##### Data Processing and Statistical Analysis

The data are presented as numbers (percentages) for categorical variables and means (standard deviation) for continuous variables. The age variable was grouped into specific age categories, and weight was categorized into three classes based on BMI. Univariate analyses were used to determine significant independent predictors of complications following TEVAR and EVAR, with corresponding odds ratios (ORs) and 95% confidence intervals (CIs). Statistical significance was set at *p* < 0.05. All data were analyzed using the Statistical Package for Software Sciences (SPSS) version 26 (IBM Corporation, Armonk, NY, USA).

### 2.3. Ethical Approval

The Institutional Review Board (IRB) of King Saud University, College of Medicine approved the study [Project No. E-22-7129]. All procedures were in accordance with the ethical standards of the institutional and national research committees, relevant guidelines and regulations, and the 1964 Helsinki Declaration and its later amendments or comparable ethical standards. The IRB waived the need for patient-informed consent because of the retrospective study design.

## 3. Results

Of the 59 patients included, 91.5% (n = 51) were men. The largest group were aged 61–70 years (n = 14, 23.7%), followed by those aged 71–80 years (n = 12, 20.3%). Additionally, 25.4% (n = 15) of the patients were under 50 years old. The numbers of overweight and obese patients were 19 (32.2%) each. Furthermore, 27.1% (n = 16) were smokers, whereas 11.9% (n = 7) were ex-smokers. The most prevalent risk factors were hypertension (n = 32, 54.2%) and diabetes (n = 25, 42.4%; Table 1).

Aneurysm repair was the most common surgery indication in 57.6% (n = 34) of patients, followed by trauma, including aortic dissections/transections, in 35.6% (n = 21). Most surgeries (64.4%, n = 38) were emergency procedures. TEVAR represented 47.5% (n = 28) of the cases, EVAR 45.8% (n = 27), and Chimney 6.8% (n = 4). Percutaneous femoral access was the predominant approach (n = 35, 59.3%). The remaining 40.7% (n = 24) required open surgical cutdown access. Medtronic endograft stents were used in 93.2% (n = 55) of patients. Most patients (81.4%, n = 48) did not develop intraoperative complications. However, endoleaks and percutaneous access failure were the most common complications, each occurring in 5.1% (n = 3) of patients. The most common intraoperative endoleak was type IA (n = 2, 66.7%). General anesthesia was performed in 79.7% (n = 47). Only nine and three patients received regional and local anesthesia, respectively (Table 2).

**Figure 1:**
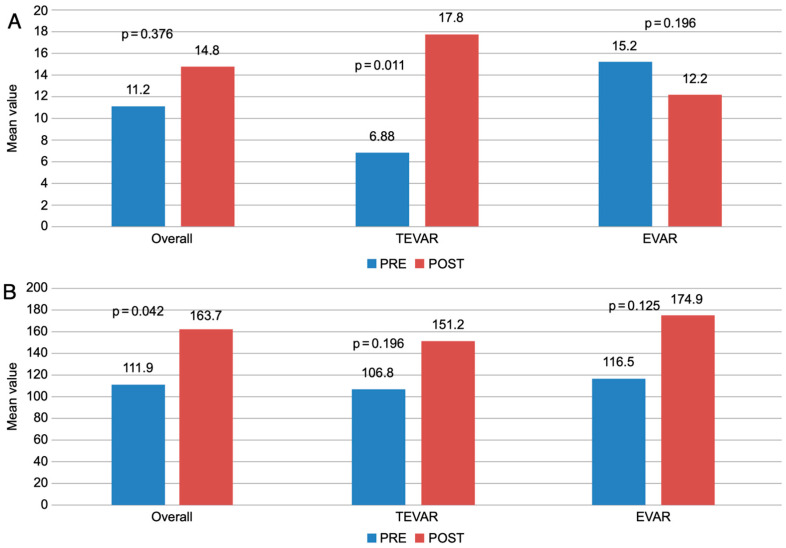
(**A**) Comparison between pre-and postoperative blood urea nitrogen and (**B**) creatinine levels. Abbreviations: BUN, blood urea nitrogen; EVAR, endovascular aneurysm repair; TEVAR, thoracic endovascular aortic repair.

The overall creatinine levels pre- and post-endovascular interventions differed significantly (*p* = 0.042). When stratifying the patients into TEVAR and EVAR groups, BUN levels increased significantly after TEVAR procedures, from 6.88 (3.27) to 17.8 (22.2) (*p* = 0.011; Figure 1A,B).

Regarding the outcome and follow-up variables, 21 (35.6%) patients required postoperative monitoring in the ICU. This relatively high rate of ICU admission can be attributed to polytrauma cases involving concomitant head and abdominal injuries. Eleven (18.6%) patients died for various reasons and were classified under all-cause mortality. After stratification into vascular-related and non-vascular-related deaths, two (3.4%) patients were determined to have died from vascular-related issues. Of these, one experienced a brainstem ischemic stroke shortly after an emergency TEVAR procedure that involved coverage of the subclavian artery origin. The other patient experienced an injury to the renal artery following a complicated Chimney procedure with subsequent intra-abdominal hemorrhage and disseminated intravascular coagulation.

Upon follow-up, 57.6% (n = 34) of patients remained stable, whereas 11.9% (n = 7) required readmission. The most common postoperative complications were endograft leaks and groin hematomas, accounting for 10.2% (n = 6) and 13.6% (n = 8) of patients. Stent thrombosis, cerebrovascular accident, and spinal cord ischemia (SCI) were reported in 5.1% (n = 3), 3.4% (n = 2), and 1.7% (n = 1) of patients, respectively. Type II endoleak was the predominant postoperative endoleak (66.7%, n = 4); two patients had type III and V endoleaks. Ten (16.9%) patients required re-intervention due to endograft leaks (60%). The average ICU stay was 2.33 days, and hospitalization lasted approximately 10.7 days (Table 3).

When comparing variables in the TEVAR and EVAR groups, it was observed that patients aged over 60 years were significantly more prevalent in the EVAR group, with 83.9% of EVAR patients being over 60 years old compared to only 32.1% in the TEVAR group (*p* < 0.001). Specifically, the 71–80 age group was well-represented in the EVAR cohort, whereas a minority of TEVAR patients fell within this age range. In contrast, the TEVAR group had a higher proportion of younger patients, with 67.9% of TEVAR patients being 60 years or younger, compared to just 16.1% in the EVAR group. The EVAR group also contained a substantially greater proportion of current/ex-smokers (*p* = 0.036). The prevalence rates of hypertension, diabetes, CAD, and coronary artery bypass grafting (CABG) were substantially higher in the EVAR group.

In the EVAR cohort, aneurysm was the primary indication in 90.3% (n = 28) of patients, whereas trauma was prevalent in the TEVAR cases (n = 19, 67.9%). Emergency surgeries were significantly higher in the TEVAR group (*p* = 0.013; Table 4).

Patients who underwent TEVAR had a significantly longer hospital stay than those who underwent EVAR. However, this could be attributed to multiple concomitant conditions, especially polytraumas (*p* = 0.012). The remaining parameters, including blood loss and operative duration, were not significantly different between the two cohorts (Table 5).

Age and aneurysmal repair have emerged as distinct and influential risk factors for TEVAR complications. The likelihood of such TEVAR complications was at least 17 times greater in patients aged >60 years than in younger patients (OR, 17.00; 95% CI, 2.262–127.7; *p* < 0.001). Patients who underwent TEVAR intervention due to an aneurysm were approximately 10.7 times more likely to experience vascular-related complications than those with traumatic injuries (OR, 10.667; 95% CI, 1.309–86.936; *p* = 0.027; Table 6). No variables were significant when predicting the risk of complications within the EVAR cohort.

## 4. Discussion

This study examined the indications, treatment outcomes, and complications of TEVAR and EVAR procedures. While OSR has historically been employed to manage aortic aneurysms, minimally invasive treatment options have gained popularity during the endovascular era [11].

Regarding the primary outcome, 11 patients (18.6%) died from various causes and were classified as having experienced all-cause mortality. Upon further stratification, two patients (3.39%) were identified as having died due to vascular-related issues. Compared with OSR, EVAR has better perioperative outcomes and lower mortality rates. However, a meta-analysis of 73 studies by Li et al. [12] revealed that EVAR is associated with higher long-term all-cause mortality, re-intervention, and secondary rupture rates than OSR. This trend persists over an extended follow-up period, with mortality rates decreasing over time. Thus, long-term monitoring is crucial for individuals who undergo EVAR. In a systematic review focusing on women in both urgent and elective settings, endovascular intervention had a lower 30-day mortality for urgent and elective AAA repair than OSR, although OSR was more frequently offered in urgent cases. However, the long-term complications were similar [13]. In contrast, previous studies have revealed that OSR has superior long-term mortality rates than EVAR, as evidenced by RCTs [8]. The 3.39% mortality rate due to vascular-related issues in this study, comparable to the rate of 4.57% after TEVAR in a previous study [14], may be attributed to several factors, including advanced age, higher American Society of Anesthesiology classification, or multiple comorbidities.

Several studies have established a correlation between EVAR, late rupture, and endograft failure [7,8], with a markedly elevated risk (four to five times) of secondary aortic aneurysm rupture compared to OSR [9]. The analysis of risk factors is crucial. In our sample, the primary demographic that underwent EVAR included overweight/obese men over 60 years old with a history of smoking and comorbidities such as hypertension and diabetes. These patterns closely mirror comparable studies [15,16], reinforcing the observed outcomes. Moreover, the mean aneurysmal diameter (6.51 ± 1.55 cm) aligns with previously published findings of 5.0–6.4 cm and 6.4 (1.3) cm for EVAR and TEVAR, respectively [12]. According to Seriki and Otoikhila, older individuals aged ≥65 years can experience a reduction in aorta elasticity, limiting its expansion [17]. This, accompanied by fatty plaque depositions in the arterial lining, increases the probability of aneurysm formation with older age [1].

In our sample, the majority of patients fell into the 61–70 years age group (23.7%), followed closely by those in the 71–80 years group (20.3%). This indicates that a significant portion of the patient population are older adults. This finding aligns with the understanding that advanced age is a common risk factor for aortic pathologies [1,2]. The demographics of the TEVAR and EVAR cohorts in our study showed that patients over 60 years of age were significantly more prevalent in the EVAR group compared to the TEVAR group (*p* < 0.001). Specifically, 83.9% of EVAR patients were older than 60 years, while only 32.1% of TEVAR patients were in this age range. This is the effect of the different causes between the groups. Specifically, TEVAR procedures were predominantly performed for traumatic cases (67.9%), whereas EVAR was primarily performed for aneurysms (90.3%). Moreover, we had 15 patients under the age of 50, representing 25.4% of the total cohort. The major reason for the inclusion of these younger patients was that they were predominantly trauma cases, which is reflected in the higher number of emergency TEVAR procedures within this age group. Similarly, Wawak et al. highlighted the outcomes of young individuals with various aortic emergencies, including aortic dissections, Takayasu disease, and fibromuscular dysplasia [18]. These findings highlight the need to pay careful attention to young individuals with aortic arch abnormalities, as they can quickly become life-threatening and necessitate immediate intervention.

Studies indicate that individuals undergoing EVAR or TEVAR, often including current and ex-smokers, exhibit reduced levels of key collagens in their arterial lining, weakening the arterial wall and causing atherosclerosis [19]. Tobacco consumption substantially increases the likelihood of developing an aortic aneurysm. Consequently, current and ex-smokers have a significant five-fold and two-fold increased risk, respectively, compared to never-smokers. Interestingly, ex-smokers, after a 25-year cessation, were at nearly the same risk level as those who never smoked [17]. In our study, the percentage of smokers/ex-smokers was considerably higher in the EVAR group. Numerous studies have demonstrated a strong correlation between smoking and the acceleration of AAAs, with a two-fold increased risk of AAA rupture [20]. According to our findings, TEVAR was primarily performed in non-smokers aged <60 years, whereas EVAR was primarily performed in smokers/ex-smokers and patients aged >60 years, consistent with a previous study demonstrating significant differences between the mean ages and smoking habits of patients who underwent these procedures [21].

Similar to a previous review [22], many patients in the EVAR cohort had hypertension, diabetes, and a history of CAD and CABG procedures, indicating a prevalent pattern of comorbidities and atherosclerosis due to advanced age.

Regarding operative details, minimally invasive percutaneous access was predominantly used (59.3%) in our study, followed by open surgical cutdown access (40.7%). Several studies have suggested a preference towards percutaneous access due to faster utility, less blood loss, and shorter hospital stays [23]. The type of stent used, and the vascular access approach could contribute to variations in the length of hospital stay. Nevertheless, the data available for analysis, particularly the numerical imbalance towards one type of material used, prevent us from concluding the effectiveness of one stent type over another.

The main complications in this study were simple groin hematomas (13.6%), followed by endograft leaks (10.2%). However, while groin hematomas were conservatively managed, endoleaks (TEVAR n = 2, EVAR n = 4) required surgical re-intervention. A previous analysis reported that endoleaks (primarily types I and III) after TEVAR occurred in 9–38% of patients, with the mean incidence being approximately 18% [24]. A high incidence of endoleaks has also been reported after EVAR procedures, with prevalence rates ranging from 10 to 40% [25]. Endoleaks warrant vigilance due to their potential threat to long-term endograft viability and an increased risk of rupture, often necessitating continuous monitoring and re-interventions [26]. In our study, 16.9% of patients required overall vascular re-intervention, the primary reason being endograft leaks.

Following groin hematomas and endoleaks, postoperative complications encompassed stent thrombosis, acute limb ischemia, and devastating neurological complications, including cerebrovascular accidents and SCI. Our study reported only a 3.57% (n = 1/28) incidence of SCI among postoperative TEVAR complications, in contrast with the 10% incidence reported previously [27].

A similar single-center study showed favorable TEVAR outcomes, including a low incidence of neurological complications, such as stroke (4.5%), SCI (1.8%), and retrograde aortic dissection (2.7%). The mortality rate was 11.7%, with instances of intraoperative conversion to OSR. The study reported a high technical success rate of 96.4% for the procedure, revealing a significant association between TEVAR and an increased need for urgent surgical intervention. These findings mirror our study’s findings, suggesting similar outcomes of TEVAR procedures [28].

The main limitation of this study was the retrospective nature of the study and the relatively small sample size. The limited cohort may have hindered the ability to detect subtle differences and associations in the data. Furthermore, since the findings of this study are based on data from a single institution, the results may not be generalizable to other settings, particularly those with different patient populations, healthcare systems, or levels of surgical expertise. Future research should prioritize multicenter studies with diverse patient populations to enhance generalizability and understand the practical outcomes of TEVAR and EVAR. Further studies are also needed to assess the feasibility of simultaneous combined procedures, such as those involving percutaneous coronary intervention [29]. Finally, the application of artificial intelligence strategies can be used to help diagnose [30], predict outcome trajectories, and define different clusters of risks [31].

## 5. Conclusions

This study highlights the efficacy and safety of TEVAR and EVAR, noting the low incidence of intra- and postoperative complications. However, despite high success and low adverse outcomes, endograft leaks remain an important concern requiring re-intervention. Further studies are encouraged to identify factors that influence the effectiveness of these procedures.

## Figures and Tables

**Table 1:** Socio-demographic characteristics of the patients (n = 59).

Study Variables	N (%)
Sex	
Women	5 (8.5%)
Men	54 (91.5%)
Age group	
18–30 years	8 (13.6%)
31–40 years	4 (6.8%)
41–50 years	3 (5.1%)
51–60 years	9 (15.3%)
61–70 years	14 (23.7%)
71–80 years	12 (20.3%)
>80 years	9 (15.3%)
BMI level	
Underweight (<18.5 kg/m^2^)	3 (5.1%)
Normal (18.5–24.9 kg/m^2^)	18 (30.5%)
Overweight (25–29.9 kg/m^2^)	19 (32.2%)
Obese (≥30 kg/m^2^)	19 (32.2%)
Smoking	
Smoker	16 (27.1%)
Ex-smoker	7 (11.9%)
Non-smoker	13 (22%)
Unknown	23 (39%)
Risk factor ^†^	
Hypertension	32 (54.2%)
Diabetes	25 (42.4%)
DLP	15 (25.4%)
CAD	14 (23.7%)
Renal insufficiency	7 (11.9%)
CHF	6 (10.2%)
CABG	6 (10.2%)
Cerebrovascular disease	5 (8.5%)
COPD	3 (5.1%)
PAD	2 (3.4%)
History of malignancy	2 (3.4%)
None	22 (37.3%)

^†^ Some patients had more than one risk factor. Abbreviations: BMI, body mass index; DLP, dyslipidemia; CAD, coronary artery disease; CHF, congestive heart failure; CABG, coronary artery bypass graft; COPD, chronic obstructive pulmonary disease; PAD, peripheral artery disease.

**Table 2:** Operative characteristics (n = 59).

Variables	N (%)
Indication for surgery	
Aneurysm	34 (57.6%)
Aortic ulcer	2 (3.4%)
Aortobronchial fistula	1 (1.7%)
Aortoesophageal fistula	1 (1.7%)
Traumatic	21 (35.6%)
Operation schedule priority	
Elective	21 (35.6%)
Emergency	38 (64.4%)
Operation type	
TEVAR	28 (47.5%)
EVAR	27 (45.8%)
Chimney	4 (6.8%)
Type of endograft	
Medtronic	55 (93.2%)
Gore	3 (5.1%)
Benteley stent	1 (1.7%)
Femoral access type	
Cutdown	24 (40.7%)
Percutaneous	35 (59.3%)
Intraoperative complications	
None	48 (81.4%)
Endoleak	3 (5.1%)
Access failure	3 (5.1%)
Renal artery injury	1 (1.7%)
Type of intraoperative endoleak (n = 3)	
1A	2 (66.7%)
1B	1 (33.3%)
Anesthesia	
GA	47 (79.7%)
Local anesthesia with sedation	3 (5.1%)
Regional	9 (15.3%)
Aneurysm diameter in cm (mean ± SD)	6.51 ± 1.55
Duration of operation in min (mean ± SD)	187.9 ± 120.3
Estimated blood loss in mL (mean ± SD)	27.9 ± 5.54

Abbreviations: EVAR, endovascular aneurysm repair; TEVAR, thoracic endovascular aortic repair; GA, general anesthesia; SD, standard deviation.

**Table 3:** Patient’s outcome and follow-up (n = 59).

Variables	N (%)
ICU transfer	
Yes	21 (35.6%)
No	38 (64.4%)
All-cause mortality	
Dead	11 (18.6%)
Alive	48 (81.4%)
Time of death (n = 11)	
30-day hospital	10 (90.9%)
>30-day hospital	1 (9.1%)
Cause of death (n = 11)	
Vascular-related	2 (3.4%)
Non-vascular-related	9 (15.3%)
Follow-up	
Stable	34 (57.6%)
Readmission	7 (11.9%)
Lost to follow-up	7 (11.9%)
Death	11 (18.6%)
Postoperative complication ^†^	
None	35 (59.3%)
Endograft leak	6 (10.2%)
Groin hematoma	8 (13.6%)
Stent thrombosis	3 (5.1%)
Acute limb ischemia	3 (5.1%)
Cerebrovascular accident	2 (3.4%)
Acute kidney injury	1 (1.7%)
Spinal cord ischemia	1 (1.7%)
Type of Postoperative endoleak (n = 6)	
2	4 (66.7%)
3	1 (16.7%)
5	1 (16.7%)
Re-intervention	
Yes	10 (16.9%)
No	49 (83.1%)
Indication for re-intervention (n = 10)	
Endograft leak	6 (60%)
Stent thrombosis	2 (20%)
Acute limb ischemia	1 (10%)
Failure of regression	1 (10%)
ICU duration in days (mean ± SD)	2.33 ± 0.58
Length of hospital stay in days (mean ± SD)	10.7 ± 8.69

^†^ Some patients had multiple postoperative complications. Abbreviations: ICU, intensive care unit; SD, standard deviation.

**Table 4:** Factors that influence operation type (TEVAR vs. EVAR) (n = 59).

Variable	Type of Surgery		*p* Value ^§^
	TEVAR N (%) (n = 28)	EVAR (including Chimney ***) N (%) (n = 31)	
Age group			
≤60 years	19 (67.9%)	5 (16.1%)	<0.001 **
>60 years	9 (32.1%)	26 (83.9%)	
Sex			
Women	3 (10.7%)	02 (06.5%)	0.661
Men	25 (89.3%)	29 (93.5%)	
BMI level			
Normal or underweight	8 (28.6%)	13 (41.9%)	0.415
Overweight or obese	20 (71.4%)	18 (58.1%)	
Smoking			
Smoker/Ex-smoker	6 (21.4%)	17 (54.8%)	0.036 **
Non-smoker	8 (28.6%)	05 (16.1%)	
Risk factor			
Hypertension	7 (25%)	25 (80.6%)	<0.001 **
Diabetes	6 (21.4%)	19 (61.3%)	0.003 **
DLP	5 (17.9%)	10 (32.3%)	0.243
CAD	2 (7.1%)	12 (38.7%)	0.006 **
CABG	0	6 (19.4%)	0.025 **
None	18 (64.3%)	4 (12.9%)	<0.001 **
Indication for surgery			
Aneurysm	6 (21.4%)	28 (90.3%)	<0.001 **
Aortic ulcer	1 (3.6%)	1 (3.2%)	
Aortobronchial fistula	1 (3.6%)	0	
Aortoesophageal fistula	1 (3.6%)	0	
Traumatic	19 (67.9%)	2 (6.5%)	
Operation schedule			
Elective	5 (17.9%)	16 (51.6%)	0.013 **
Emergency	23 (82.1%)	15 (48.4%)	
Femoral access			
Cutdown	15 (53.6%)	09 (29%)	0.068
Percutaneous	13 (46.4%)	22 (71%)	
ICU transfer			
Yes	14 (50%)	7 (22.6%)	0.034 **
No	14 (50%)	24 (77.4%)	
Mortality			
Dead	6 (21.4%)	5 (16.1%)	0.741
Alive	22 (78.6%)	26 (83.9%)	
Intraoperative complication *			
Yes	2 (7.1%)	4 (12.9%)	0.673
No	26 (92.9%)	27 (87.1%)	
Postoperative complication			
Yes	6 (21.4%)	13 (41.9%)	0.105
No	22 (78.6%)	18 (58.1%)	
Re-intervention			
Yes	2 (7.1%)	8 (25.8%)	0.084
No	26 (92.9%)	23 (74.2%)	

^§^ *p*-value has been calculated using Fischer’s Exact test. * One patient experienced more than one intraoperative complication. ** Significant at *p* < 0.05 level. *** The chimney technique in endovascular aortic aneurysm repair (Ch-EVAR) involves placement of a stent or stent-graft parallel to the main aortic stent-graft to extend the proximal or distal sealing zone while maintaining side branch patency. Abbreviations: BMI, body mass index; CAD, coronary artery disease; CABG, coronary artery bypass grafting; DLP, dyslipidemia; EVAR, endovascular aneurysm repair; ICU, intensive care unit; TEVAR, thoracic endovascular aortic repair.

**Table 5:** Factors that influence the operation type (TEVAR vs. EVAR) (contd.) (n = 59).

Factor	Type of Surgery		*p* Value ^‡^
	TEVAR Mean ± SD (n = 28)	EVAR Mean ± SD (n = 31)	
Diameter of an aneurysm in cm	6.8 ± 1.96	6.44 ± 1.48	0.614
Duration of operation in minutes	169.1 ± 104.9	205 ± 132.1	0.256
Estimated blood loss in mL	178.6 ± 161.8	288.7 ± 281.3	0.074
Length of hospital stay in days	13.7 ± 9.78	8.1 ± 6.68	0.012 **
Highest postoperative BUN	17.8 ± 22.2	12.2 ± 10.9	0.214
Highest postoperative creatinine	151.2 ± 194.7	174.9 ± 243.6	0.683

^‡^ *p*-value was calculated using an independent sample *t*-test. ** Significant at *p* < 0.05 level. Abbreviations: BUN, blood urea nitrogen; EVAR, endovascular aneurysm repair; SD; standard deviation; TEVAR, thoracic endovascular aortic repair.

**Table 6:** Determining significant independent predictors of complication after TEVAR operation (n = 28).

Factor	OR	95% CI	*p* Value
Age group			
≤60 years	Ref		
>60 years	17	2.262–127.7	<0.001 **
Smoking			
Smoker/Ex-smoker	Ref		
Non-smoker	0.071	0.005–1.059	0.055
Hypertension			
Yes	5.667	0.890–36.085	0.066
No	Ref		
Diabetes			
Yes	3.400	0.516–22.406	0.203
No	Ref		
Indication for surgery			
Aneurysm	10.667	1.309–86.933	0.027 **
Traumatic	Ref		
Operation schedule			
Elective	Ref		
Emergency	0.529	0.070–3.978	0.537
ICU transfer			
Yes	1.000	0.056–17.751	1.000
No	Ref		

Abbreviations: CI, confidence interval; ICU, intensive care unit; OR, odds ratio. ** Significant at *p* < 0.05 level.

## Data Availability

The data are available from the corresponding author upon reasonable request.
